# ACE inhibitor induced intestinal angioedema

**DOI:** 10.1111/ans.17575

**Published:** 2022-02-23

**Authors:** Tran Ngoc An Huynh, Lina Hua, Johan Andries Smalberger, Justin James

**Affiliations:** ^1^ Department of General Surgery Box Hill Hospital Melbourne Victoria Australia; ^2^ Department of Anatomy The University of Melbourne Melbourne Victoria Australia; ^3^ Imaging Associates Box Hill Melbourne Victoria Australia; ^4^ Faculty of Medicine, Nursing and Health Sciences Monash University Melbourne Victoria Australia

A 44‐year‐old Caucasian female presented to an Emergency Department (ED) of a large metropolitan hospital with a one‐day history of severe abdominal pain after commencing oral perindopril the day prior.

Her medical history included hypertension and a laparoscopic appendicectomy. She had no medication allergies, and no travel history, dietary or environmental exposure of concern.

She was commenced on oral perindopril 4 mg daily by her family doctor for hypertension. After an hour of taking the first dose of perindopril, she began feeling extremely lethargic. The next morning, after 30 min of taking the second dose, she developed intermittent non‐radiating abdominal cramps. By the afternoon, she felt nauseated, vomited five times and had one episode of non‐bloody diarrhoea. Her abdominal pain progressed to severe grade, which prompted her to present to ED.

On the initial examination, she was afebrile and appeared well. There were no skin rashes, lip or tongue swelling. Her abdomen was soft with generalised tenderness, most pronounced in the central abdomen.

Her white cell count was mildly elevated at 12.7 × 10^9^/L. The rest of her blood counts, serum electrolytes and renal and liver function tests were normal. Beta hCG was negative. Computed tomography (CT) of the abdomen and pelvis showed moderate ascites and bowel wall thickening with submucosal oedema about the duodenum and proximal jejunum. There was mild oedema of the small bowel mesentery (Fig. 1).

**Fig. 1:**
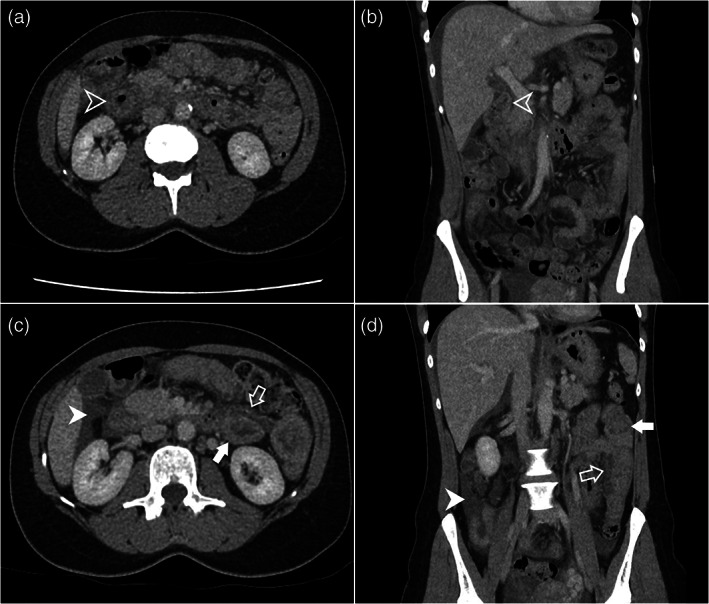
(a) Axial and (b) coronal computed tomography show duodenal thickening with submucosal oedema (empty arrowhead) with periduodenal oedema. (c) Axial and (d) coronal computed tomography, in the same patient, show small bowel wall thickening with submucosal oedema (solid arrow), and adjacent mesenteric oedema (empty arrow), as well as ascites (solid arrowhead).

Due to the close temporal relationship with the new medication, angiotensin‐converting enzyme inhibitor (ACEi) induced intestinal angioedema was suspected, and the ACEi was ceased. The patient improved with simple analgesia, intravenous fluid therapy and bowel rest. She was discharged home 3 days later after the complete resolution of her symptoms. She was commenced on amlodipine 2.5 mg daily for the management of hypertension.

She was followed up in the allergy outpatient clinic 10 days after discharge. Her complement levels, flow cytometry, C1 esterase inhibitor function (C1‐INH), and tryptase level were normal, keeping with an ACEi induced angioedema. She was discharged to her family doctor with the advice of lifelong ACEi avoidance.

ACEi are widely used in managing hypertension and other cardiovascular diseases. However, they are adversely associated with angioedema, with an overall incidence of 0.1–0.7%.^1^ ACEi are well‐known to cause oedema of the face, tongue, lips and upper airways. Intestinal angioedema is much rarer and less well known.^2^ Angioedema commonly occurs within the first 6 weeks of starting ACEi therapy. However, onset can vary from hours to years.^3^

The mechanism of ACEi induced angioedema is postulated to be related to increased levels of bradykinin.^4^, ^5^ The vasodilatory effects lead to increased capillary permeability and extravasation of fluid, resulting in angioedema.^3^

Clinically, the patient may experience diffuse abdominal pain, nausea, vomiting, and diarrhoea. Complement and C1‐INH levels should be measured during the symptomatic period to increase diagnostic yield.^3^ Abnormal complement levels may indicate hereditary or an acquired form of angioedema, whereas normal levels would be in keeping with ACEi induced angioedema.^3^, ^6^

On CT, the radiographic signs include circumferential small‐bowel wall thickening, mesenteric oedema and ascites.^7^ Duodenal involvement is rare, but commonly affects the jejunum, followed by the ileum.^8^, ^9^

These patients often pose diagnostic dilemmas as their non‐specific symptoms can mimic an acute abdomen. A 2011 literature review found that 57% of cases had surgery or gastrointestinal biopsies.^9^ Thus, it is important for surgeons to have an early clinical suspicion of ACEi induced intestinal angioedema.

ACEi induced angioedema is treated by elimination of the causative agent.^4^ After elimination of the ACEi, improvement of symptoms usually occurs within 12–72 h, which subsequently confirms the diagnosis.^4^, ^6^ Patients only require supportive care, with no need for antihistamines or corticosteroids.^3^, ^4^, ^6^ Follow‐up care is essential to establish a formal diagnosis and ensure close monitoring of an alternative agent.^3^ A rechallenge with an ACEi is strongly discouraged.^3^

ACEi induced intestinal angioedema should be strongly considered when evaluating a patient with non‐specific abdominal pain who is concurrently receiving ACEi therapy. Thus, a thorough medication history is important when evaluating cases of unclear aetiology of intestinal angioedema. Early recognition of ACEi induced intestinal angioedema avoids unnecessary and invasive interventions.

Informed consent was obtained from the patient.

## Author contributions

**Tran Ngoc An Huynh:** Conceptualization; data curation; writing – review and editing. **Lina Hua:** Writing – review and editing. **Johan Andries Smalberger:** Data curation; investigation; writing – review and editing. **Justin James:** Conceptualization; writing – review and editing.
